# Adequate Oxygenation State Maintained during Electroconvulsive Therapy in Nonobese Patients Using the Oxygen Reserve Index: A Pilot Study

**DOI:** 10.1155/2023/7807693

**Published:** 2023-11-06

**Authors:** Yuji Kadoi, Jo Ohta, Yumeka Sasaki, Shigeru Saito

**Affiliations:** ^1^Division of Operation Room, Gunma University Hospital, Maebashi, Japan; ^2^Department of Anesthesiology, Gunma University School of Medicine, Maebashi, Japan; ^3^Department of Psychiatry, Gunma University School of Medicine, Maebashi, Japan

## Abstract

Some controversial reports have observed oxygen desaturation (defined as percutaneous oxygen saturation (SpO_2_) < 90%) during electroconvulsive therapy (ECT). The purpose of this pilot study was to examine oxygenation states in eight patients during ECT. In addition to the usual hemodynamic monitors and pulse oximeter, the oxygen reserve index (ORi) was monitored using a pulse oximeter. Patients received either no preoxygenation or preoxygenation with 100% oxygen via a tight-fitting mask for 1 or 3 min before induction of anesthesia. ORi increased after preoxygenation. ORi differed significantly between 3 min of preoxygenation and the other two methods before restarting mask ventilation. SpO_2_ was significantly increased with all methods before stopping manual mask ventilation or before restarting manual mask ventilation compared with that before preoxygenation. No oxygen desaturation was observed at any time with any treatment methods. In nonobese patients, the adequate oxygenation state as shown by SpO_2_ and ORi was maintained during ECT even without preoxygenation.

## 1. Introduction

Electroconvulsive therapy (ECT) lasts only a few minutes, so manual mask ventilation is a standard procedure [1–5]. Various reports have described frequent oxygen desaturation (defined as percutaneous oxygen saturation (SpO_2_) < 90%) during ECT [1–5]. Umamaheswara Rao et al. [1] reported oxygen desaturation in 29% (93 of 316 patients) during ECT, with this frequency increasing to 54% in obese patients (BMI ≥ 30 kg/m^2^; vs. 27% in nonobese patients) and 37% in patients with prolonged seizure duration (≥45 s; vs. 26% in patients with seizure duration <45 s). However, those patients were not preoxygenated prior to induction of anesthesia for ECT. Other reports have shown frequencies of desaturation ranging from 2.5% to 27.5% [1–5]. In contrast, Zhu et al. [3] found no desaturation during ECT. Such contradictory results might be attributable to differences in anesthetic management during ECT. Anesthetic management, particularly manual mask ventilation and preoxygenation, could greatly influence the oxygenation state during ECT. In clinical anesthetic management, oxygen desaturation is seldom observed during the short duration of apnea in the intubation period (1-2 min) in cases provided with several minutes of preoxygenation and manual mask ventilation during the induction of anesthesia [6]. As the no-ventilation time (i.e., the period without manual mask ventilation) during ECT should be relatively short (≤2 min), desaturation would seem unlikely during ECT in nonobese patients. We question the frequent observation of desaturation during ECT reported by McCormick and Saunders [2] and Umamaheswara Rao et al. [1]. Several minutes of preoxygenation before induction of anesthesia are widely known to prevent desaturation during apnea (the period of intubation management) [6]. We therefore hypothesized that few instances of a desaturation state would be observed after several minutes of preoxygenation before anesthesia during ECT in nonobese patients. The purpose of this pilot study was to examine oxygenation states during ECT.

## 2. Case Presentations

Eight patients who underwent ECT between April 2021 and August 2021 were enrolled in this study. All the eight patients underwent ECT during this period.

Informed consent was obtained from patients or their families. All protocols were approved by the institutional clinical study committee (approval no. HS2021-178). None of the patients were obese (body mass index (BMI) ≥ 30 kg/m^2^) or had any history of respiratory, cardiovascular, cerebrovascular, hepatic, renal, or neuromuscular disease.

### 2.1. Anesthetic Management

Eight patients (mean age, 49 ± 6 years; mean height, 159 ± 3 cm; mean weight, 60 ± 7 kg; BMI, 24 ± 3 kg/m^2^) undergoing ECT were enrolled in this study. No obese patients were encountered because of the lower BMI in Japanese populations than in Western populations. All the patients underwent at least 10 sessions of ECT (3 times per week at 1- or 2-day intervals).

Blood pressure (BP), heart rate, SpO_2_ as measured by pulse oximetry from the left or right index finger, end-expiratory partial pressure of carbon dioxide (end-tidal CO_2_(PetCO_2_)) at the nostrils (Capnomac Ultima™; Datex Co., Helsinki, Finland), and electrocardiographic parameters from lead II were measured during the procedure.

In addition to the usual pulse oximeter, the oxygen reserve index (ORi) was monitored using a Radical-7^R^ pulse oximeter (Masimo Co., Irvine, CA) to ensure oxygenation remained adequate during the absence of manual mask ventilation [4]. Measurements were initiated prior to the induction of anesthesia and were terminated at the end of the ECT procedure.

Preoxygenation was performed for 1 or 3 min before the induction of anesthesia using 100% oxygen delivered via a tight-fitting mask. In the third session of ECT, 1 min of preoxygenation was performed (Group A), and in the fourth session of ECT, 3 min of preoxygenation was performed (Group B). Patients were also monitored in the absence of preoxygenation (Group C).

After preoxygenation (0, 1, or 3 min), anesthesia was induced using propofol at 1.0 mg/kg; then, 0.6 mg/kg of suxamethonium was administered. After inducing anesthesia, hyperventilation (expired end-tidal carbon dioxide (PetCO_2_) of approximately 30 mmHg) was achieved by tight manual mask ventilation delivering 100% oxygen. When muscular relaxation was judged as adequate, bilateral electrodes were placed, and the electrical stimulus for ECT was delivered (Thymatron®System IV; Somatics LLC, Lake Buff, IL) via bifrontal-temporal electrode placement.

Manual mask ventilation was stopped immediately before electrical stimulus and then restarted immediately after seizure cessation on electroencephalography (EEG). No manual mask ventilation or provision of oxygen was performed during the EEG seizure.

### 2.2. Statistical Analysis

All data are expressed as the mean ± standard deviation. Repeated-measures analysis of variance (ANOVA) was used for comparisons of the time course of changes in ORi among each group, and Bonferroni testing was used for multiple comparisons between groups at each measurement time point. Values of *p* < 0.05 were considered statistically significant. Calculations were performed using StatView version 5.0 software (Abacus Concepts, Berkeley, CA).

Groups showed no significant differences in EEG seizure duration (Group A (1-min preoxygenation), 43 ± 17 s; Group B (3-min preoxygenation), 47 ± 19 s; Group C (no preoxygenation), 43 ± 6 s; *P* = 0.85) or time from stopping manual mask ventilation to restarting manual mask ventilation (i.e., no-ventilation time) (Group A, 70 ± 29 s; Group B, 88 ± 52 s; Group C, 86 ± 19 s; *P* = 0.61). Groups also showed no significant differences in time from starting manual mask ventilation to stopping manual mask ventilation immediately before electrical stimulus (i.e., manual mask ventilation time) (Group A, 82 ± 17 s; Group B, 89 ± 18 s; Group C, 102 ± 13 s; *P* = 0.20). Table 1 shows changes in SpO_2_ and ORi over time in the three groups. ORi was increased after 1 or 3 min of preoxygenation. ORi was significantly greater in Group B (3 min of preoxygenation) than in the other groups just before restarting mask ventilation. SpO_2_ was significantly higher in all groups both before stopping manual mask ventilation and before restarting manual mask ventilation compared with before preoxygenation.

## 3. Discussion

This study failed to identify any periods of desaturation (SpO_2_ < 90%) during ECT in nonobese patients, with adequate oxygenation (SpO_2_ > 96%) maintained during ECT even without preoxygenation.

The frequency of oxygen desaturation during ECT remains controversial [1–5]. Such contradictory results might be attributable to differences in inspired oxygen concentration, use of preoxygenation, and provision of supplemental oxygen during the seizure using a nasal cannula. In addition, Lew et al. [5] described the use of manual mask ventilation during the seizure to prevent hypoxemia. However, manual mask ventilation during the seizure could plausibly exert a strong influence in the precise recording of EEG. It is thus advisable for anesthesiologists to stop manual mask ventilation during EEG recording to avoid introducing artificial EEG noise and ensure precise measurement of the EEG seizure duration. This in turn makes it essential that the patient reaches a sufficiently oxygenated state prior to the no-ventilation time (the period of EEG recoding) during ECT.

Preoxygenation is often used to prevent desaturation during the induction of anesthesia [6]. We therefore hypothesized that the use of preoxygenation could maintain adequate oxygenation during ECT. The mean EEG seizure duration in this study was 45 s, longer than suggested by Umamaheswara Rao et al. [1]. No-ventilation time (time from stopping manual mask ventilation to restarting manual mask ventilation) was 85 s in this study. No desaturation state was found during the no-ventilation period even when no preoxygenation was used. Our findings suggest that adequate oxygenation as shown by SpO_2_ can be maintained during the short period of apnea in ECT even when manual mask ventilation is started after anesthesia induction without preoxygenation in nonobese patients. In addition, 90 seconds of manual mask ventilation before electrical stimulation may have been enough to prevent desaturation during the no-ventilation period when neither mask ventilation nor provision of supplemental oxygen was performed during the seizure.

To assess the adequacy of oxygenation during the no-ventilation period without manual mask ventilation, we used ORi in addition to a standard pulse oximeter. Ishida et al. [7] reported that monitoring of ORi can allow the detection of a hypoxemic state earlier than standard pulse oximetry. A decrease in ORi is reportedly observed about 30 s earlier than a decrease in standard pulse oximetry [7]. ORi thus appeared useful for detecting hypoxemia earlier during the no-ventilation period when manual mask ventilation was not being performed during the seizure. Even when preoxygenation was not performed, the oxygenation state was sufficient to avoid hypoxemia (SpO_2_ < 90%). Although ORi was maintained in the three groups during the observation period, ORi in the group with 3 min of preoxygenation was higher than in the other two groups before restarting manual mask ventilation. In contrast, no difference in ORi was seen between no-preoxygenation and 1-min preoxygenation groups during the observation period. These findings suggest that at least 3 min of preoxygenation is needed in cases where the oxygenation state may be expected to decrease during the no-ventilation period.

The decrease in ORi after the seizure might be attributable to the increased oxygen demand caused by the seizure, while no decrease in SpO_2_ was observed. Oxygen demand reportedly increases 1.5- to 2-fold compared with that in the preseizure period [8].

The present study did not examine the oxygenation state in obese patients or patients with obstructive sleep apnea, who appear more susceptible to developing desaturation during ECT [9, 10]. Future work should therefore clarify the oxygenation state in obese patients. In addition, we used 0.6 mg/kg of suxamethonium to induce anesthesia as a dose that produced tracheal intubation conditions as satisfactory as those with suxamethonium at 1.0 mg/kg [11, 12] while decreasing the incidence of desaturation due to minimized oxygen consumption. Tang et al. [13] reported that use of succinylcholine at 1.5 mg/kg was associated with significantly more rapid desaturation than rocuronium at 0.9 mg/kg for anesthesia induction because of increased oxygen consumption caused by succinylcholine-related skeletal muscle fasciculations. Such findings suggest that different results might be observed when using larger doses of suxamethonium for anesthesia induction.

In conclusion, no desaturation state was observed during ECT, and adequate oxygenation (SpO_2_ > 96%) was maintained during ECT even when preoxygenation was not performed for nonobese patients.

## Figures and Tables

**Table 1:** Time course of changes in ORi and SpO_2_ between the three groups.

	①	②	③	④	⑤	⑥	⑦
ORi							
No preoxygenation	0	NA	NA	0.52 ± 0.07	0.42 ± 0.16	0.38 ± 0.18	0.31 ± 0.06
One minute	0	0.11 ± 0.24	0.27 ± 0.36	0.49 ± 0.26	0.44 ± 0.24	0.40 ± 0.22	0.36 ± 0.28
Three minutes	0	0.10 ± 0.23	0.38 ± 0.32	0.60 ± 0.26	0.61 ± 0.26^*∗*^	0.44 ± 0.31	0.43 ± 0.30
SpO_2_ (%)							
No preoxygenation	95 ± 1.0	NA	NA	99 ± 0.6^#^	99 ± 0^#^	98 ± 0.5#	98 ± 0.6
One minute	95 ± 1.9	96 ± 1.8	98 ± 1.6	99 ± 0.9^#^	99 ± 0.9^#^	98 ± 1.5	98 ± 1.4
Three minutes	95 ± 1.4	97 ± 1.0	99 ± 1.0	99 ± 0.7^#^	99 ± 0.5^#^	99 ± 1.1	99 ± 0.7

Data are expressed as mean ± SD. ORi: oxygen reserve index; NA: not assigned. One minute: one minute of preoxygenation before induction of anesthesia. Three minutes: three minutes of preoxygenation before induction of anesthesia. ① Before preoxygenation; ② thirty seconds after starting oxygenation; ③ one minute after starting oxygenation; ④ before cessation of manual mask ventilation; ⑤ before restarting manual mask ventilation; ⑥ at time of restarting spontaneous breathing; ⑦ full recovery of spontaneous breathing. ^*∗*^*p* < 0.05 compared with the other two groups. ^#^*p* < 0.05 compared with the preoxygenation period.
